# Local Plasticity of Dendritic Excitability Can Be Autonomous of Synaptic Plasticity and Regulated by Activity-Based Phosphorylation of Kv4.2

**DOI:** 10.1371/journal.pone.0084086

**Published:** 2014-01-03

**Authors:** Anna Labno, Ajithkumar Warrier, Sheng Wang, Xiang Zhang

**Affiliations:** 1 Nano-scale Science and Engineering Center, Berkeley, California, United States of America; 2 Biophysics Program, University of California Berkeley, California, United States of America; Institut National de la Santé et de la Recherche Médicale (INSERM U901), France

## Abstract

While plasticity is typically associated with persistent modifications of synaptic strengths, recent studies indicated that modulations of dendritic excitability may form the other part of the engram and dynamically affect computational processing and output of neuronal circuits. However it remains unknown whether modulation of dendritic excitability is controlled by synaptic changes or whether it can be distinct from them. Here we report the first observation of the induction of a persistent plastic decrease in dendritic excitability decoupled from synaptic stimulation, which is localized and purely activity-based. In rats this local plasticity decrease is conferred by CamKII mediated phosphorylation of A-type potassium channels upon interaction of a back propagating action potential (bAP) with dendritic depolarization.

## Introduction

Persistent modifications of neuronal function, in response to repetitive and precisely timed synaptic stimuli are believed to be the key mechanism underlying learning, memory formation and storage [1]. While these modifications are based on the modulation of synaptic strengths [2], it is widely believed that different forms of synaptic plasticity can alter local dendritic excitability by modulating both resting and voltage-gated channels along the length of the dendrites [3], [4] and that such compartmentalized dendrites can greatly expand the computational power of a single neuron [5]. In contrast to alterations of global excitability, which may occur independently of synapses, localized modulations of dendritic excitability have never been observed in the absence of synaptic plasticity [6]–[9]. Kv4.2 channels play crucial role in controlling neuronal excitability by mediating transient A-type potassium currents [10], [11], have been directly associated with spatial memory in rats [12] and are implicated in a number of hyperexcitability and neurodegenerative diseases such as epilepsy [11], [13]–[15], ischemia [16], [17] and Fragile X mental retardation [18], [19]. Up to date dendritic patch clamp recordings were used to study localized changes of dendritic excitability. However it is challenging to use dendritic recordings to study localized excitability in multiple different cellular compartments of the same cell with high spatiotemporal resolution due to difficulty of patching more than a couple of cellular sites at the same time and inability to relocate the patch site, thus leaving key questions about the role of dendritic excitability in plasticity unsolved. Is dendritic excitability contingent upon synaptic processes or can dendrites detect activation patterns independently? What role do active dendrites play in memory storage and in facilitating synaptically based storage? What mechanisms regulate Kv4.2 channel phosphorylation and localization?

## Materials and Methods

### Cell culture and Transfection

Animal euthanasia procedures were conducted according to guidelines approved by the Office of Laboratory Animal Care (OLAC) Committee on Laboratory and Environmental Biosafety University of California, Berkeley, which approved this study. Animals (neonatal rats) are obtained from the Animal facility, and decapitated after brief carbon dioxide anesthesia. Hippocampi were dissected from P1-2 Sprague Dawley rats of either sex, and kept in ice-cold HEPES buffered Hanks' Balanced Salt Solution (HBSS, GIBCO) at all times. Cells were dissociated with trypsin for 10 min at 37°C, followed by gentle trituration. The dissociated cells were then transfected with pcDNA3.1/hChR2-EYFP (kind gift from Karl Deisseroth, sequence can be found in the every Vecotr depository (http://www.everyvector.com/sequences/show_public/2498) to allow for transient photodepolarization of dendritic membrane [20]–[23] using Nucleofector-II (Amaxa Biosystems) in accordance with manufacturer's protocol (1) and plated at a density of 25,000–50,000/cm2 on poly-l-lysine-coated glass coverslips. Dissociated neurons were cultured in Neurobasal medium (GIBCO) supplemented with B-27 (Invitrogen) and penicillin-streptomycin (10U/ml, GIBCO). Experiments were done on morphologically identified pyramidal neurons 14–18 d in vitro (DIV).

### Optical Setup

**Figure 1:**
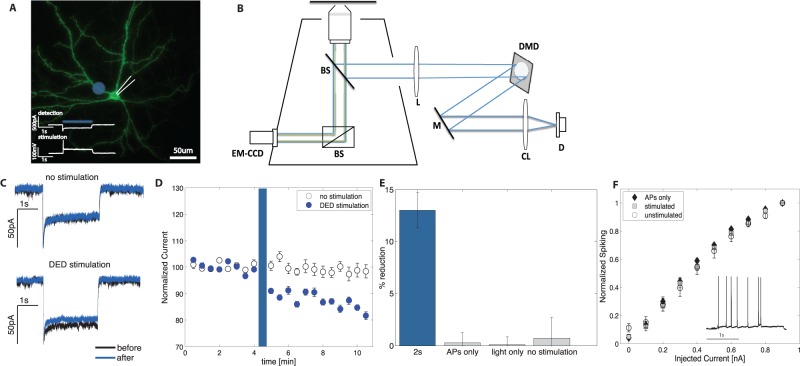
Persistent decrease in dendritic excitability following paired stimulation. (**A**)EYFP-ChR2 expressing hippocampal neuron is stimulated with ∼28 um light spot (blue circle) applied on the proximal dendrites coupled with somatically induced spike. The insert shows the detection protocol (top) where 2 s photostimulation was used to determine dendritic excitability and stimulation protocol (bottom) where the same 2 s photostimulation of proximal dendrite is coupled with a spike (**B**) Schematics of the optical setup. Patterned ChR2 stimulation is achieved by a 470 nm LED is collimated by a lens (CL) and directed using a mirror (M) to Digital Micromirror Device (DMD). The light reflected from DMD is collimated and scaled using a lens system (L) and coupled into the microscope via a beam splitter (BS). (**C**) An example trace for 2 s report photo depolarization before (black trace) and after (blue trace) the treatment. When no stimulation was applied (top) no change in dendritic excitability is observed, however after paired stimulation (bottom) excitability decreased (**D**) Dendritic excitability prior to paired stimulation, shows a steady basal level of dendritic excitability as assessed by measuring the peak magnitude of the ChR2-induced photo current. After paired stimulation the current decreases by 13% for 2 s report as compared to 0.74% for no stimulation. (**E**) Only stimulation by paired APs and dendritic depolarization causes DED = 12.6% (p<0.05)). Controls, where no stimulation takes place or either APs or light alone are delivered show no significant DED (p>0.05 in all cases). (**F**) There is no change in spike-current relationship between stimulated (□) and unstimulated neurons (o) or neurons stimulated only with APs (▴) indicating no change in intrinsic excitability. The excitability was measured by applying depolarizing current pulses (2 s in duration) in 0.1-nA increments. For each depolarizing step, the number of evoked action potentials was counted and plotted against injected current amplitude. Inset shows a representative current clamp trace.

**Figure 2:**
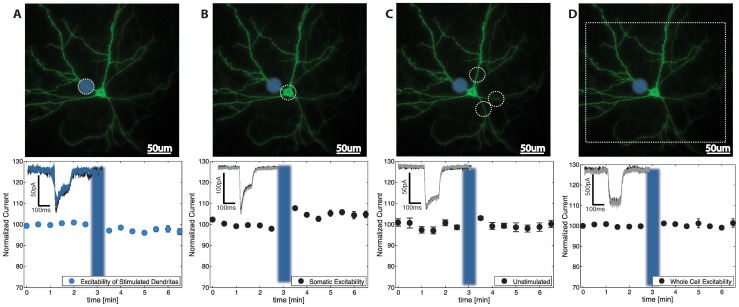
DED is spatially localized. (A, B, C and D) Top panels display the dendritic location used for dendritic stimulation (blue circle) and the range of possible report locations (white dotted lines). Bottom panels show example current measurements before (black) and after stimulation (grey or blue) as well normalized current before and after stimulation. Following proximal dendritic stimulation, (A) proximal dendritic current decreases (DED = 2.94%±2.19%, p<001, n = 11/12) (B) somatic current shows increase in excitability by 4.89%±1.89 (p<0.001 n = 10/10) (C) Un-stimulated dendrites don't show DED (%DED = 0.05%±2.93%, p = 0.22, n = 10/10) and (D) Current resulting from whole cell photo-stimulation does not change significantly (DED = −0.4%±1.9, n = 9/9, p = 0.10).

Hippocampal neurons were placed in a perfusion chamber and visualized using inverted Nikon TE-2000E microscope and Andor EM-CCD (Andor). The cell plane was illuminated with X-Cite 120 lamp (Lumen Dynamics) and only neurons, which expressed EYFP, were chosen for experiments. To generate patterned illumination, a 470 nm LED (Phillips) was expanded, collimated and reflected directly from digital mirror device (DMD, InFocus LP435Z) coupled into the microscope (Fig. 1 B). The diode was synchronized with electrical stimulation through a TTL signal to provide step on/off light stimulus and DMD was controlled using VGA signal from a computer. DMD patterns were generated via custom written MATLAB (Mathworks) software, which allowed user to position an arbitrary light pattern over the displayed cell image. For a majority of the experiments, a circular pattern of 28 um in diameter was positioned over the imaged proximal section of the dendrite. Somatic and whole cell measurements (shown in Fig. 2 B and D) were measured analogously to dendritic excitability but rather than photostimulating the dendrite we photo-stimulated soma or the entire cell respectively. A set of previously determined affine transformations were applied to the pattern, so that after passing through the optical path of the microscope, it would be correctly positioned with respect to the cell. To stimulate the cell we paired thirty 2s photocurrent injections (or 100 ms in case of data presented in Figure 2) at 0.2 Hz which caused sub-threshold depolarization, into proximal dendritic compartment, with APs (20 ms after the onset of the light), which were evoked by depolarizing the cells to approximately +40 mV for 10 ms.

### Electrophysiology

Individual coverslips were placed in a recording chamber (Biosciences) and submerged in room temperature ACSF (145 mM NaCl, 3 mM KCl, 10 mM HEPES, 20 mM glucose, 2 CaCl_2_ and 1 MgCl2) supplemented with 0.1 mM picrotoxin (Sigma), 0.01 mM DNQX (Sigma) at 322 mOsm, pH 7.4. Voltage or current clamp recordings were performed in the perforated somatic patch-clamp configuration. Electrodes (2–5 MΩ) were pulled from borosilicate glass tubing (World Precision Instruments) using a laser based micropipette puller (Sutter Instrument P-2000). The pipette tip was dipped in an intracellular solution containing (in mM) 68 K-gluconate, 68 mM KCl, 0.2 mM EGTA, 2 MgSO4, 20 HEPES, 3 ATP, 0.2 mM GTP (322 mOsm, pH 7.4) and then backfilled with the same solution but containing 0.12 mg/ml amphotericin B (Sigma); final osmolarity was about 290 mOsm/kg H_2_O and open pipette resistance when filled was 2–5 MΩ. A stable perforated patch was obtained after 15–20 min incorporation time. The access resistance was ∼15–40 MΩ and to minimize the effect of access resistance change on measured current we only recorded cells which showed small variations of access resistance (less than 5%) through the entire experiment. Currents were recorded using patch-clamp amplifier Axopatch 200B-2 in conjunction with Digitizer 1440A, sampled at 10 kHz. The holding potential in voltage-clamp mode was −70 mV, uncorrected for any liquid junction potential between internal and external solutions. All the experiments were conducted in voltage clamp except the DED stimulation and measurement of spike-current relationship, which was conducted in current clamp. Intrinsic excitability was measured by applying 2 s depolarizing current pulses in increasing current increments (0.1-nA increments) and at each depolarizing step, the number of evoked action potentials was counted and plotted against injected current amplitude [24]. Recordings were analyzed using pClamp v10 software or custom written Matlab code. Whenever % decrease is mentioned in the text it refers to the difference between averaged excitability over the entire duration of the experiment before and after stimulation – typically 5–10 min. For pharmacological experiments all drugs were obtained from Sigma-Aldrich with an exception of Stromatotoxin-II which was obtained from Alome Labs. All drugs were bath applied and handled according to manufacturer recommendations.

### Immunofluorescence staining of neurons

Dendritic excitability decrease was induced as described in the methods section above with the exception that DED stimulation was Thirty trains of stimuli were delivered using Iso Flex Unit Stimulator (A.M.P.I) rather than patch pipette (AMPI Jerusalem, Israel). Two elongated electrodes delivered these 10 ms stimuli to the culture. The voltage drop across the preparation was approximately 70V and we confirmed that this stimulus was capable of robustly inducing spiking. Cells were fixed immediately after stimulation with 4% paraformaldehyde (Sigma) in PBS for 20 minutes before being permeabilized (1% Triton-X, Sigma) and blocked in block solution (PBS containing 5% BSA). Then cells were incubated for 4 h with anti-pKv4.2 Ser 438 monoclonal antibodies (Santa Cruz Biotechnology) and then Alexa Fluor 568 secondary antibodies (Invitrogen). Coverslips were mounted using Fluoromount-G (SouthernBiotech) and sealed with nail polish. Cells were visualized using a Nikon TE-2000 inverted epifluorescence microscope through 60×0.8 NA Nikon or 100×1.3NA Zeiss objective. Images were digitally captured using EMCCD Andor. Excitation was via X-Cite lamp with appropriate filter cubes. Images were analyzed using ImageJ and custom written Matlab (Mathworks) software.

### Data Analysis

**Figure 3:**
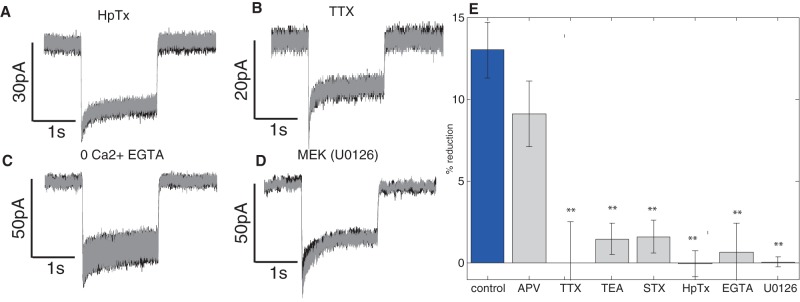
Kv4.2 channels are responsible for DED and the process requires Ca2+ and MEK. (A–D) Example current measurements before (black) and after stimulation (grey) showing that DED is blocked by HpTx (A) and TTX (B). Moreover DED does not happen in the absence of Ca2+ (C) and is blocked by MEK inhibitor U0126 (D). (E) Bar graph summarizing effect of different drugs. When no drugs are applied 13%±1.7% DED is observed. Application of TTX (1 uM), TEA (20 mM) and Stromatotoxin-II (100 nM) reduced the amount of DED to ∼3% while application of Hptx (100 mM) or U0126 (10 uM) or removal of calcium from external solution completely abolishes DED. The dendritic excitability was measured using 2 s pulses and the DED was quantified as a percentage change between the averaged peak current before and after stimulation. For each treatment significance of differences between the excitability before and after the stimulation was tested with two tailed heteroscedastic Student's t-test. Comparison between conditions was conducted by one-way ANOVA with Tukey post hoc test and showed no significant difference between control and APV (p = 0.08) and a statistically significant difference between control and all other conditions with p<0.001.

The dendritic excitability was assessed by measuring the peak of the photocurrent at each stimulation over time [8], [10] except for the first two stimulations in each experiment, which were removed because at that time ChR2 hasn't reach steady state desensitization level. The data were normalized by taking the averaged excitability measured before the stimulation to be 100% and then normalizing each indyvidual excitability measurement (both before and after stimulation) to that. Significance of differences between the excitability before and after the stimulation was tested with two tailed heteroscedastic Student's t-test, except of the spike-current data and pharmacological data (Fig. 3), which were tested using ANOVA test with Tukey post hoc comparison, and p<0.05 was considered as significant. Throughout the paper dendritic excitability changes are given as mean ± standard error on the mean and n = no/no indicates number of cells/number of experiment; asterisk, p<0.05; double asterisk, p<0.01; triple asterisk, p<0.001. The images of immunostaining against Kv4.2 phosphoylated at Ser 438 were analyzed by first normalizing all the images to account for differences in light intensity by dividing each pixel in the image by its average intensity. Then the image of the dendrite was straighten using ImageJ Plugin (Straighten, [25]) and average fluorescence along the dendrite was calculated by drawing a line through the center of the dendrite and calculating average along this line. In each cell, stimulated dendrite and another dendrite with comparable amount of YFP staining were chosen for analysis. If there weren't any unstimulated dendrites that have similar amount of YFP as the simulated one the cell was not used for analysis. Significance of differences between the fluorescence in stimulated and unstimulated dendrites was tested with unpaired Student's t-test.

## Results

To understand the contribution of activity to plasticity of dendritic excitability we developed a technique to decouple the dendritic excitability changes from synaptic strength changes by stimulating dendrites with a protocol similar to spike timing dependent plasticity (STDP) substituting presynaptic cell stimulation with localized photostimulation of ChR2 using a digital micromirror device (DMD). The use of a DMD allowed us to photostimulate multiple sub-cellular locations simultaneously and to vary the locations of the depolarizations with millisecond resolution (Fig. 1.a,b) [26]. Synaptic transmission was completely blocked by α-amino-3-hydroxy-5-methyl-4-isoxazolepropionic acid (AMPA) and gamma-aminobutyric acid (GABA) receptor antagonists (10 uM DNQX and 100 uM PTX).

### Persistent change in dendritic excitability can be induced independently of synaptic inputs

We observed that even in the absence of synaptic inputs, pairing thirty 2s photocurrent injections into proximal dendritic compartment, with APs (20 ms after the onset of the light) resulted in a persistent (>15 min) decrease in dendritic excitability as indicated by the decrease of amplitude of evoked current at the soma (Fig. 1c–d, DED = 13%±1.7% p<0.001, n = 12/12, no stimulation  = 0.74%±2.0% p = 0.35 n = 6/6). We will refer to this phenomenon as dendritic excitability depression (DED). DED requires a coincident interaction of dendritic stimulation and APs and neither APs alone (DED = 0.28%±0.98% p = 0.68, n = 7/7) nor dendritic stimulation alone (DED = 0.1%±0.76%, p = 0.88, n = 6/6) induced DED. Similarly no stimulation of any kind also does not result in a change of excitability (0.74%±2.0% p = 0.35 n = 6/6, Fig. 1e). This suggests that dendrites can detect coincident dendritic stimulation and APs independently of synaptic AMPA and GABA receptors. Because DED can only be induced by coincident dendritic depolarization and AP and not by light stimulation alone shows that the change in dendritic excitability is not a result of photo damage due to repetitive photo stimulation. We observed no change in intrinsic, whole-cell excitability accompanying DED, as indicated by unchanged Spike-Current relationship (Fig. 1f, p = 1.00, n = 3/3). This suggests that DED was the result of localized dendritic excitability modulation.

### DED is spatially localized

To investigate how different sub-cellular compartments respond to DED we induced DED at one of the proximal sites and measured the excitabilities at other dendrites, soma and excitability of the entire cell. In this experiment we used 100 ms stimulation to determine dendritic excitability because prolonged 2s photo-stimulation injected too much current when applied on the cell body or over the entire cell causing abnormal spiking. First we stimulated a proximal dendrite and observed a change of excitability on that dendrite, just as before, but using 100 ms photo-stimulation. DED was smaller in magnitude but still reliably induced (Fig. 2a, DED = 2.94%±0.63%, p<0.001, n = 11/12). When the whole cell was photo-stimulated the excitability was not affected (DED = −0.13%±1.0%, n = 10/10, p = 0.98, Fig. 2d), supporting the observation that intrinsic excitability does not change. On the other hand the somatic excitability, measured by somatic current, increased (Fig. 2b increase of 5.23%±0.98%, p<0.001, n = 10/10). Finally unstimulated dendritic branches were unaffected by the stimulation of another branch (Fig. 2b. %DED = 0.05%±0.93%, p = 0.46, n = 10/10). Taken together, these results show that DED is confined to a stimulated dendrite and coupled with modulation of somatic excitability, which might, akin to homeostatic plasticity, serve to keep overall neuronal excitability unchanged [27].

### Kv4.2 channels are responsible for DED

Since voltage-gated channels regulate dendritic processing by dynamically modulating membrane excitability in a spatially restricted manner [28], [29], we tested their involvement in DED. Application of non-selective voltage-gated potassium channel inhibitor (20 mM TEA) eliminated most of the DED (DED = 1.47%±0.95%, p = 0.08, n = 10/10, Fig. 3e). Stromatotoxin (100 nM), which specifically inhibits delayed rectifier and A-type potassium channels [30] (Kv2.1, Kv4.2, Kv2.2 and Kv2.1/9.3), significantly reduced DED (Fig. 3e, DED = 1.6%±1.0%, p = 0.01, n = 5/5). To distinguish between Kv2.1 and Kv4.2 channels, we bath-applied Heteropodatoxin-2 (Hptx, 100 nM) which specifically blocks Kv4.2, Kv4.1 and Kv4.3 but not Kv2.1 [31]. Hptx completely eliminated DED (Fig. 3a and e. DED = −0.04%±0.79%, p = 0.92, n = 10/10). This data indicates that decrease of dendritic excitability is conferred by changes in A-type potassium currents mediated by Kv4.2 channels.

Since Kv4.2 channels also control bAPs, we further investigated whether bAPs are essential for induction of DED. If an interaction between bAPs and dendritic stimulation is required for DED, then it is reasonable to assume that abolishing spikes by bath application of sodium channel blockers should reduce DED [32], [33]. Application of 1 uM tetrodotoxin (TTX) precluded induction of DED (Fig. 3b,e. DED = 0%±2.5%, p = 0.84, n = 5/5), suggesting that DED requires coincident interaction of bAPs and dendritic depolarization.

Subsequently we wanted to investigate if calcium, which is essential for many neurological processes, is necessary for DED. To determine this we attempted to induce DED in 0 Ca2+ with 2 mM EGTA-AM, which can be passively loaded into cells to chelate intracellular calcium. Dendritic excitability didn't change with calcium buffered (DED = 0.65%±1.8%, p = 0.33, n = 7/7). Then we turned our attention to MEK, which because MAPK cascade is known to be able to integrate coincident signals and to translate the magnitude of signaling into a temporally and spatially graded response [34] and has been previously implicated in learning and memory in behaving animals [35] and shown to be necessary for many forms of synaptic plasticity [34], [36] and dendritic excitability regulation [37] although its precise role is unknown Blocking MEK using 10 uM of U0126 abolished induction of DED (DED = 0.07%±0.3%, p = 0.93).

Finally, to ensure that DED is not a result of synaptic process where NMDA receptors are activated by local depolarization and Glu released from synapses or ambient Glu – released from other synapses – in the cleft we induced DED in the presence of NMDA blocker APV (50 uM). Dendritic excitability decreased 9.1%±2.0% (p<0.001, n = 5/5) indicating that NMDA receptors are not involved in DED and that the process is independent of synaptic receptors.

Taken together this data suggest DED stimulation induces interaction of bAP with local dendritic depolarization, which may increase the level of intracellular calcium. Elevated calcium activates a signaling cascade involving MEK, which then affects the Kv4.2 channels leading to an increased A-type current and decreased excitability.

### MEK regulated CamKII phosphorylation of Kv4.2 underlies DED

**Figure 4:**
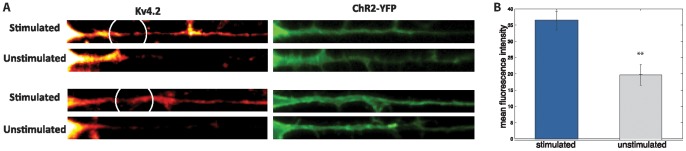
Kv4.2 phosphorylation is enhanced along the stimulated dendrite. (A) Immunostaining against Kv4.2 phosphoylated at Ser 438 shows enhanced phosphorylation at the stimulated dendrite as compared with the un-stimulated dendrite of the same cell. The dendrites have comparable amounts of ChR-YFP. Dendrites from two representative cells are shown (top and bottom). The white circles indicate the location of photostimulation. (B) Mean immunofluorescence of pKv4.2 along the stimulated dendrites is much higher (mean fluorescence  = 36.5±2.84) than along the non-stimulated dendrites (mean fluorescence  = 19.8±3.17), p = 0.01, n = 5/5.

To gain further insight into the physical mechanism, that allows Kv4.2 channels to modulate dendritic excitability dynamically, we performed immunostaining against Ser 438 phosphorylated Kv4.2. Ser438 is the site that CamKII specifically phosphorylates Kv4.2 at. The density of phosphorylated Kv4.2 along the stimulated dendrite was significantly higher (Fig. 4, increase 16.7%, p = 0.01, n = 5/5) than along a comparable, unstimulated dendrite on the same cell. This suggests that DED is associated with differential phosphorylation of Kv4.2. This phosphorylation increases Kv4.2 current [38] and may also affects translocation direction and turnover rate of Kv4.2 [39], [40].

## Discussion

Activity dependent modulations in dendritic excitability are central to information processing and storage but so far have only been seen in addition to synaptic plasticity. Here we show that localized depression of dendritic excitability can be decoupled from synaptic processing. DED cannot be induced by dendritic photo stimulation alone or APs alone indicating that this change in dendritic excitability is not an artifact of photo stimulation resulting in channel damage or persistent somatic stimulation, but rather it is a persistent physiological change brought about by coincided dendritic activity.

DED is confined to the stimulated dendrite and brought about by interaction of bAPs with dendritic depolarization. This coincidence is detected in NMDA-independent way, possibly via PKC pathway, which has been previously proposed to regulate excitability [41] and serve as a coincidence detector [42], [43]. Since DED is calcium dependent and cannot be induced in the absence of calcium we hypothesize that this interaction induced increase in intracellular calcium, which results in MEK-regulated phosphorylation of Kv4.2 at Serine 438 residue. MEK is known to regulate Kv4.2 phosphorylation by activating either ERK [44] or CamKII [45], [46] which can then directly phosphorylate Kv4.2. Inhibition of MEK interferes with LTP induction [37], [47]. CamKII phosphorylates Kv4.2 at Ser438 while ERK phosphorylates Kv4.2 at T602, T607, and S616. DED is accompanied by increased levels of Kv4.2 phosphorylated at Ser438 at the stimulated dendrite, suggesting that increased calcium causes ERK activation, which in turn activates CamKII which directly phosphorylates Kv4.2. Ser438 Kv4.2 phosphorylation leads to increase in local cellular Kv4.2 and potentiation of A-type current [38] and hence decreases dendritic excitability by approximately 12%. During STDP LTP induction blocking MEK activity reduced the boosting of the action potential by a similar amount [47].

While previously redistribution of A-type potassium channels was shown to accompany various forms of LTP [11], [28], [38] here we show for the first time that Kv4.2 channels can self-organize to locally alter the excitability of the dendrite in the absence of any signaling resulting from synaptic potentiation. Recently, compartmentalized branch specific potentiation of dendritic excitability has been demonstrated following repeated local spike initiation with transient application of carbachol or theta pairing protocol [8]. In contrast to DED this stimulation protocol resulted in potentiation of branch strength which might be due to different stimulation protocol or likely involvement of synaptic plasticity [48]. Since A-type K^+^ currents are the major modulator of back-propagating action potentials (bAP) [49] and increase in A-type current, such as one that could be causing DED, decreases bAP [50], [51], DED may decrease both the bAP [49]–[51] and forward propagating sub-threshold photocurrent. This would lead to depression of dendritic current reaching the soma but also limits further development of DED by decreasing the magnitude of bAPs. Such compartmentalized and active excitability modulation can lead to forming privileged and repressed pathways of activity and may be a general feature of dendritic information storage and would greatly increase neuronal storage capacity [52], [53]. It suggests that dendrites can play a far more crucial and independent role than previously believed by self-organizing in response to activity rather than being synaptically controlled.
